# Atypical IgG4‐related disease limited to the sino‐nasal cavity: A case report

**DOI:** 10.1002/ccr3.4207

**Published:** 2021-05-19

**Authors:** Ahmed Shaikh, Karol Silla, Adham A. Aljariri, Mouhammad Zuhair Sharaf Eldean, Hamad Al Saey

**Affiliations:** ^1^ Otolaryngology Department Ambulatory Care Center (ACC) Hamad Medical Corporation (HMC) Doha Qatar; ^2^ Pathology Department Hamad General Hospital (HGH) Hamad Medical Corporation (HMC) Doha Qatar

**Keywords:** fibroinflammatory disorders, IgG4‐related disease, rhinology, sino‐nasal

## Abstract

Given the overlapping clinical features of sino‐nasal immunoglobulin 4‐related disease (IgG4‐RD) to rhinitis or rhinosinusitis, this paper aims to delineate this rare, isolated manifestation significant to physicians for their daily practice and researchers contributing to this field.

## Graphical abstract

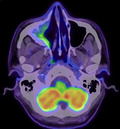

## 1 BACKGROUND

We are presenting a case report about sino‐nasal immunoglobulin 4‐related disease (IgG4‐RD) in a middle‐aged Southeast Asian man without systemic disease.

IgG4‐related disease (IgG4‐RD) refers to a group of immune‐mediated inflammatory disorders with overlapping characteristic features clinically and on histopathology.^1^, ^2^ This systemic condition tends to be benign and chronic inflammatory sclerosing in nature is infiltrated by positively staining IgG4‐positive plasma cells.^3^, ^4^, ^5^, ^6^ This infiltrate also leads to fibrosis organized in a storiform pattern with mild‐to‐moderate tissue eosinophilia or that which is obliterative in nature.^7^ Two common findings associated with this disease are allergic conditions and tumefactive lesions, which can manifest highly destructive behavior involving bony invasion, nerve infiltration, and bone marrow infiltration.^1^, ^3^, ^4^, ^8^ In terms of treatment, there is a favorable prognosis if treated early with prednisone.^3^, ^4^, ^9^ Moreover, a prospective, open‐label clinical trial and case series reported the effectiveness of rituximab for IgG4‐RD even without adjunctive glucocorticoid therapy.^10^

Typically, multiple organs are involved and could include the lacrimal gland, salivary gland, thyroid gland, pancreas, biliary tract, and retroperitoneum. ^4^, ^6^, ^7^, ^11^, ^12^ On the other hand, IgG4‐RD presenting as an isolated sino‐nasal lesion is extremely rare in the literature.^3^, ^13^ Facial pain, epistaxis, and the clinical symptoms of chronic rhinosinusitis were commonly described in the sino‐nasal mucosal presentation of this IgG4‐RD.^14^ Furthermore, in the first systematic review of IgG4‐RD presentation in the head and neck that reviewed 484 patients, who majority were from Asia, however, none were reported from the Middle East or India.^15^ This case report is about sino‐nasal IgG4‐RD in a middle‐aged Southeast Asian man without systemic disease.

## 2 CASE PRESENTATION

A 38‐year‐old Indian male patient, with no known comorbidities, was referred to the ENT outpatient clinic due to persistent right‐side nasal pain and frequent nasal bleeding that stops with conservative measures, the patient did not have visual symptoms, he denied any systemic symptoms as arthralgia, fever, weight loss, or generalized weakness, the patient gave a history of endoscopic sinus surgery in the right nasal side done abroad 6 months prior to the presentation, and histopathology showed sino‐nasal IgG4‐related disease.

The physical examinations showed absent inferior nasal turbinate, crusts, and adhesions in the right nasal cavity with a clear left nasal cavity.

**FIGURE 1:**
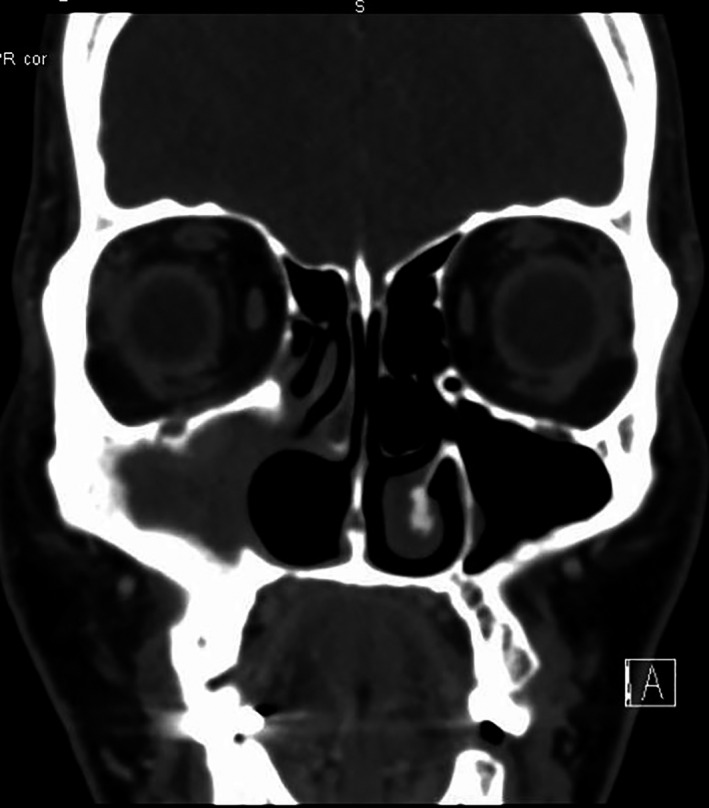
Sinus CT scan with contrast showing postoperative changes of uncinectomy and medial maxillectomy

**FIGURE 2:**
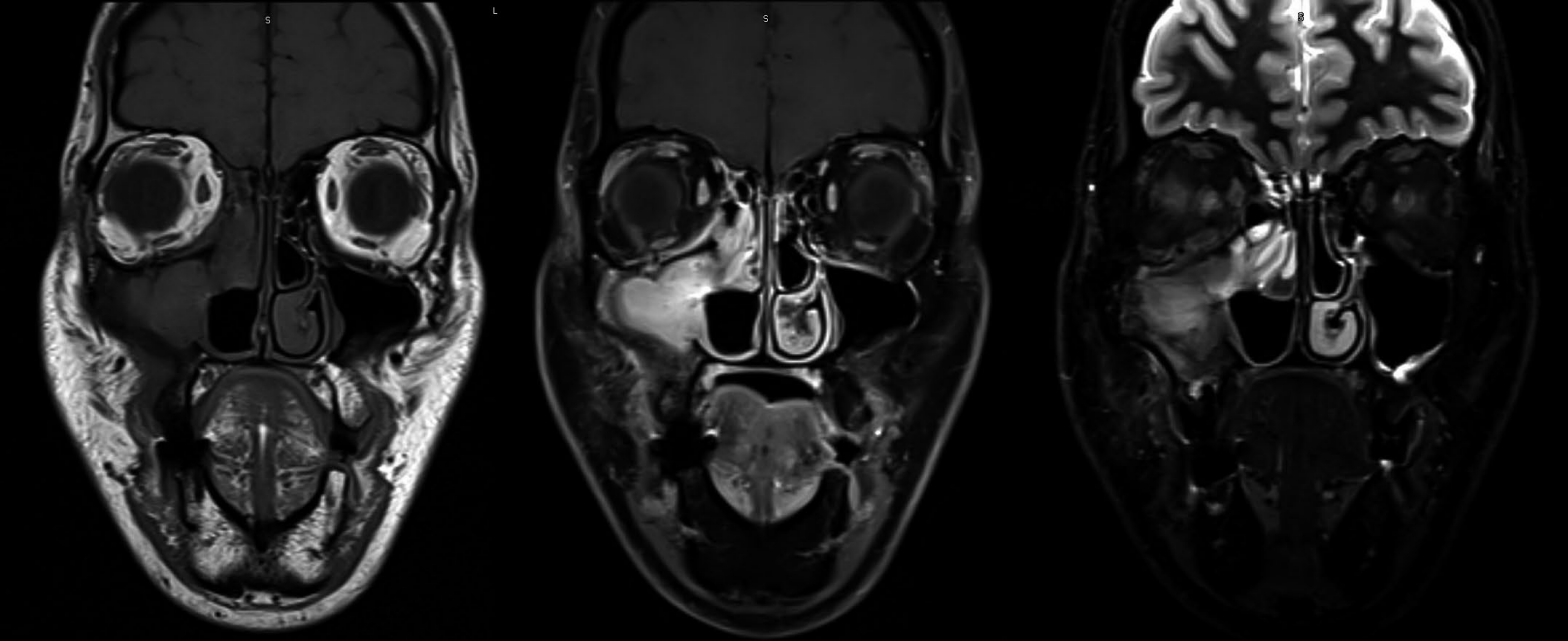
T1, T1 with and T2 with contrast MRI showing enhancing mass lesion in the right maxillary sinus

The lesion evaluated radiologically by Sinus CT (computed tomography) scan with contrast, Sinus MRI (magnetic resonance imaging) with contrast (Figures 1,2) which showed evidence of previous right uncinectomy, medial maxillectomy, and right inferior turbinectomy, with mass occupying the right maxillary sinus and destruction of lamina papyracea without signs or orbital herniation.

The patient was referred to the rheumatology team to check for rheumatologic manifestations and systemic involvement, clinically patient did not have any complaints, blood workup showed high blood levels of IgG4 257.00, reference range (3.00‐201.00), total IgE of 976.00 Reference range (0.00‐114.00), and blood flow cytometry showed a low level of CD19 count of 1.00 reference range (107.00‐698.00).

**FIGURE 3:**
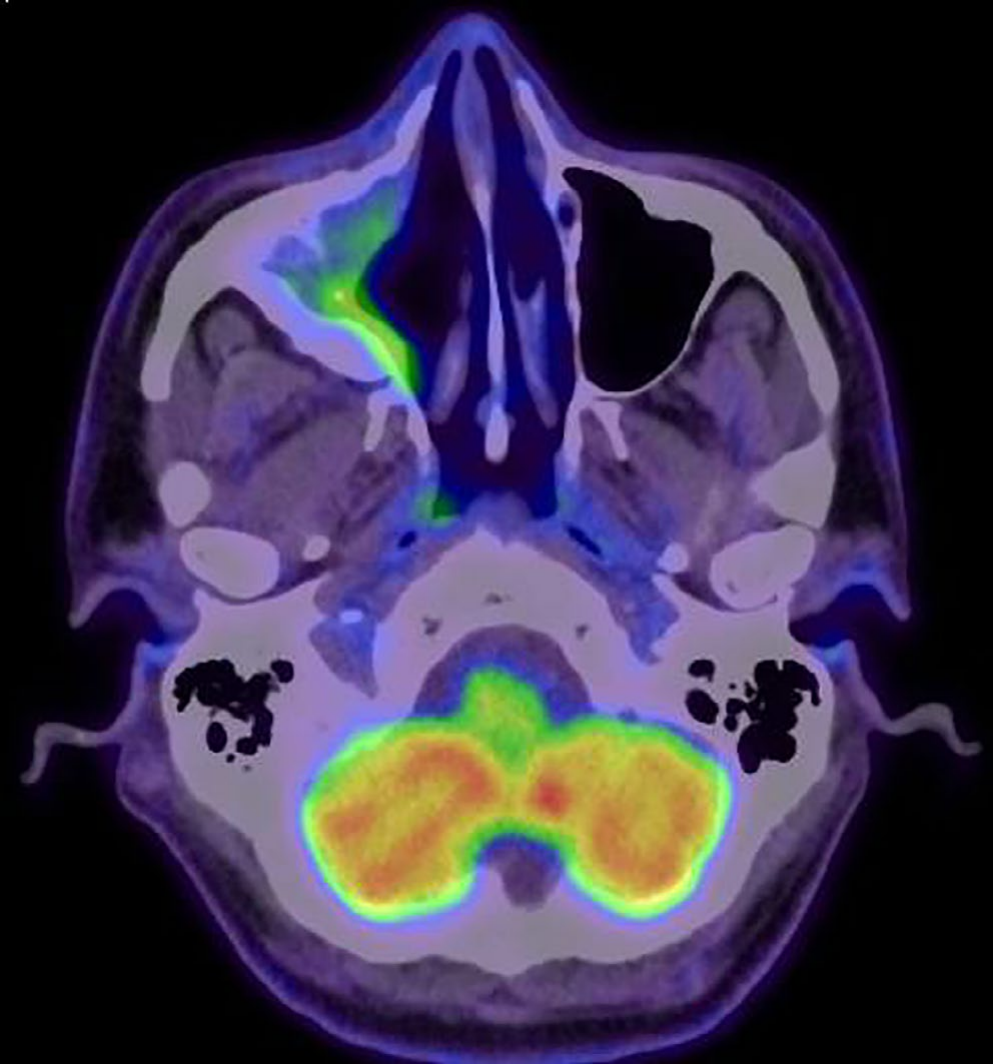
FDG PET CT scan showing axial cut of the sinus with metabolic activity limited to the right maxillary sinus walls

The patient underwent FDG PET CT scan (fluorodeoxyglucose‐positron emission tomography‐computed tomography; Figure 3) that showed increase metabolic activity in the walls of right maxillary sinus with no signs of distant metabolic activity, and the patient was cleared from rheumatological point of view and diagnosed as isolated right sino‐maxillary IgG4 disease.

**FIGURE 4:**
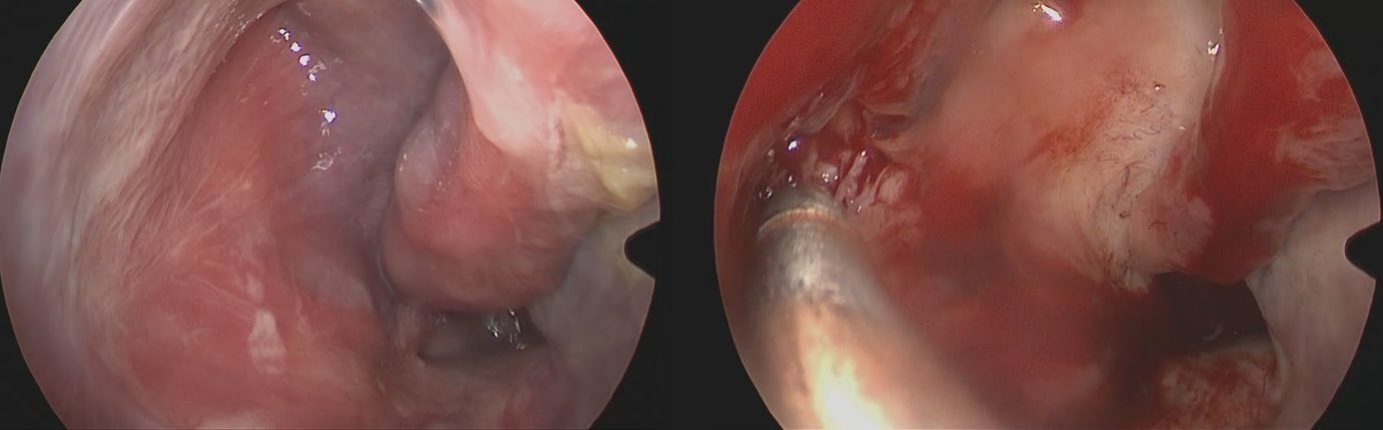
Intraoperative image of the mass, right image shows mass occupying the right maxillary sinus, left showing mass excision with shaver

The patient underwent mass debulking and excision (Figure 4) then he was followed by rheumatology team where he received three doses of rituximab (each dose of 1 gram) and low dose prednisolone 5 mg daily for 3 months then tapered to 2.5 mg over the same period, the patient is following with rheumatology team on 3‐month bases and disease progression is followed clinically and with lab tests.

The patient was followed by the ENT team on regular basis every 3 months, he did not give any history of recurrent epistaxis, and physical examination showed no signs of local recurrence.

## 3 HISTOPATHOLOGY

Sections of the nasal biopsy show upper respiratory mucosa overlying fibrotic soft tissue infiltrated by numerous plasma cells and scattered small lymphocytes.

**FIGURE 5:**
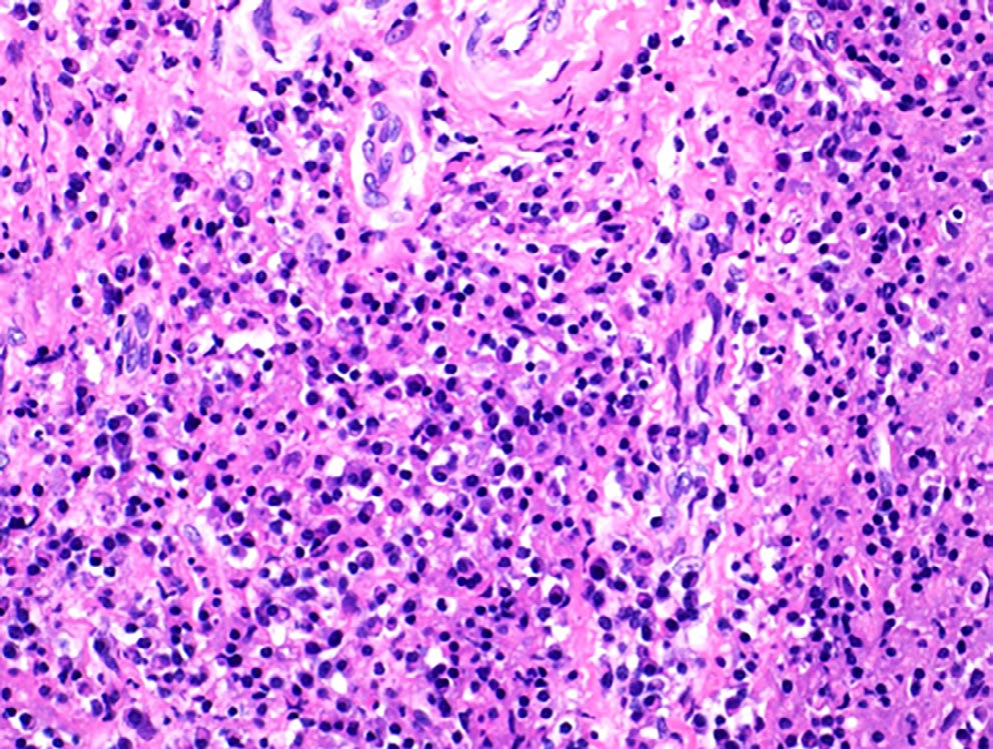
High power magnification of H&E‐stained biopsy from the nasal cavity showing fibrous tissue infiltrated by mature‐looking plasma cells

**FIGURE 6:**
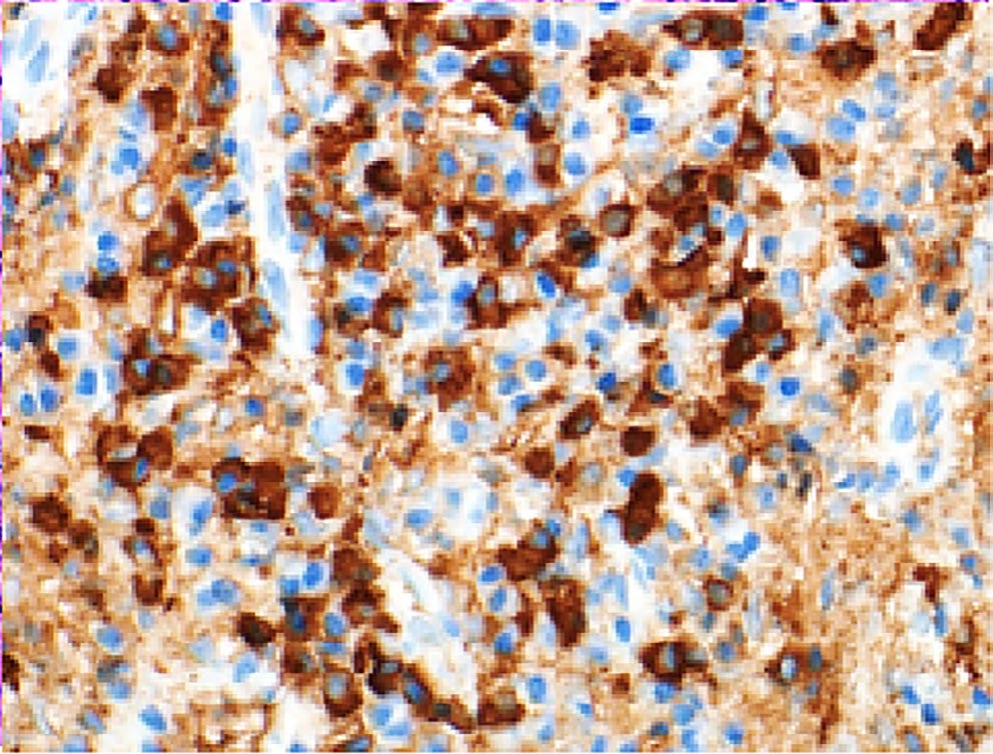
Immunohistochemical stain showing IgG4 in the mass

**FIGURE 7:**
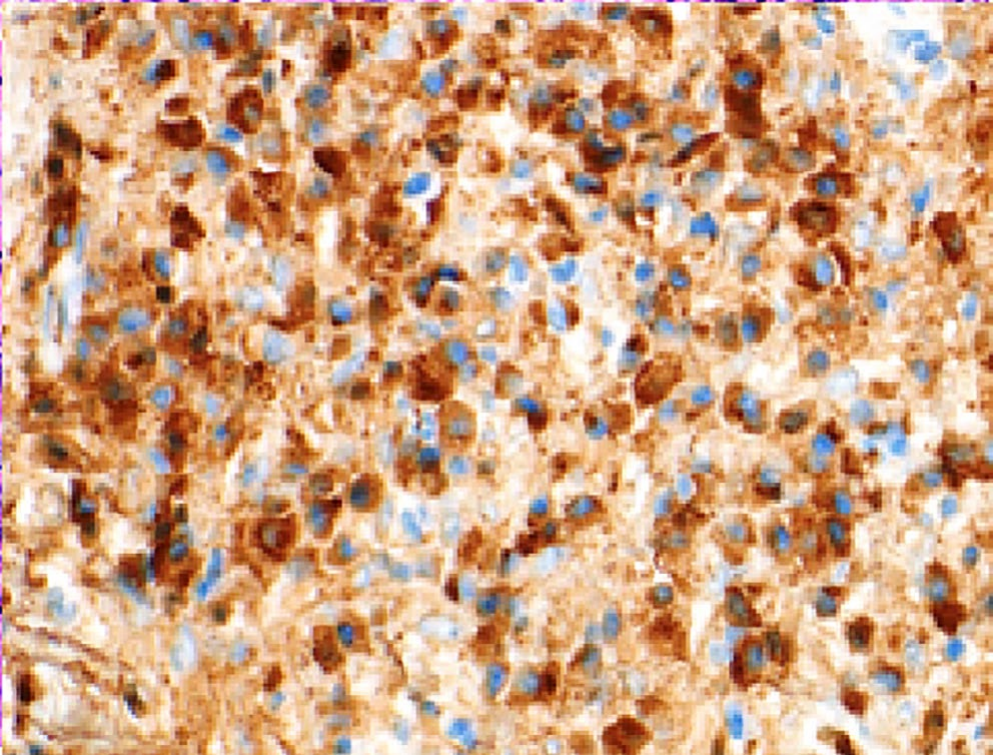
Immunohistochemical stain showing IgG in the mass

By immunohistochemical stains, the plasma cells are highlighted by CD138 (Figure 5) and show polyclonal staining with Kappa and Lambda in situ hybridization (ISH). The ratio of IgG4 positive cells (Figure 6) to IgG positive cells (Figure 7) is high (above 40%).

Note the significantly increased IgG4 to IgG ratio.

## 4 DISCUSSION

Currently, the majority of the patients reported in the literature come from the United States and Japan.^15^ Interestingly, a recent cross‐sectional study that gathered cases of IgG4‐RD head and neck presentation among patients from America, Europe, and Asia reported a predilection for female patients and patients of Asian descent.^16^ It is common among middle‐aged, elderly men above 50 years old.^6^ Our patient happens to come from Southeast Asia. It would be worth investigating what etiology puts certain populations at risk or whether a higher index of clinical suspicion exists in certain parts of the world compared to other regions. ^15^

A literature review that investigated eight patient cases showed that IgG4‐RD frequently involved the maxillary sinus; six of those had a local extension to the sino‐nasal area. After urgently ruling out common malignant sino‐nasal tumors like squamous cell carcinoma and lymphoma, this disease must be high in differential not to delay diagnosis and treatment. ^17^

Currently, no definitive diagnostic criteria for IgG4‐RD affecting the sino‐nasal cavity are established; rather, a 3‐tiered diagnostic algorithm in a consensus statement was proposed during the first international symposium on IgG4‐RD in 2011.^18^ This statement declares that the diagnosis of IgG4‐RD is highly suggestive or probable depending on which histopathology features are met, including “dense lymphoplasmacytic infiltration with increased IgG4‐positive plasmacytosis, fibrosis, often storiform in character, and obliterative phlebitis.”^19^ Our patient falls under the category of “histologically highly suggestive of IgG4‐RD” after the meeting.^13^

Symptoms mimicking rhinitis or rhinosinusitis including facial pain due to pressure, bloody nasal discharge, and nasal obstruction may prompt prescription of steroid sprays in the clinic, possibly delaying or missing investigating for IgG4‐RSD, which is only an emerging disease in the literature.^15^ Moreover, despite the diagnosis of rhinosinusitis, there are cases reported of a patient with rhinosinusitis complicated with IgG4‐RD (Table 1), so this differential must always be kept in mind.^20^

**TABLE 1:** Available literature review of IgG4‐related disease sino‐nasal manifestations

	Author, Year	Title	Age, gender (M; male, F: female), nationality/race if indicated	Location
1.	Ishida, M., et al	Multiple IgG4‐related sclerosing lesions in the maxillary sinus, parotid gland, and nasal septum	72, M, Japanese	Maxillary sinus, parotid gland and nasal septum
2.	Pace, C., et al	A rare case of IgG4 related sclerosing disease of the maxillary sinus associated with bone destruction	73, M	Maxillary sinus extending to infratemporal fossa
3.	Ikeda, R., et al	A case of paranasal sinus lesions in IgG4‐related sclerosing disease	50, F	Maxillary and ethmoid sinus
4.	Sasaki, T., et al	Immunoglobulin G4 − Related Sclerosing Disease Mimicking Invasive Tumor in the Nasal Cavity and Paranasal Sinuses	71, M	Nasal cavity, maxillary sinuses, pterygopalatine fossa, and sphenoid
5.	Lindau, R. H., et al	Immunoglobulin G4‐related sclerosing disease of the paranasal sinus	69, M	Maxillary sinus to orbit
6.	Alt, J. A., et al	Locally destructive skull base lesion: IgG4‐related sclerosing disease	38, F	Sphenoid sinus
7.	Morris, C., et al	Immunoglobulin G4 related disease isolated to the nasal cavity: a rare cause of nasal obstruction	34, M	Sinonasal cavity (nasal septum and lateral nasal wall)
8.	Song, B. H., et al	A rare and emerging entity: Sinonasal IgG4–related sclerosing disease	72, M	Maxillary and ethmoid sinuses extending to orbital cavity
9.	Dosen, L. K., et al	IgG4‐Related Nasal Pseudotumor	34, M, Caucasian34, F, Sri Lankan	Nasal cavity pseudotumor
10.	Kurien R, et al	Unusual cause of maxillary sinus mass with proptosis	21, M, Indian	Mass in left maxillary sinus, orbit, infratemporal fossa and anterior cranial fossa
11.	Inoue, A., et al	IgG4‐related disease in the sinonasal cavity accompanied by intranasal structure loss	70, M	Posterior ethmoid, maxillary sinus, nasal turbinates
12.	Gontarz, M., et al	IgG4‐related disease in the head and neck region: report of two cases and review of the literature	30, M	Maxillary sinus, ethmoid sinuses, cervical lymph nodes, and upper gingiva
13.	Vandjelovic, N. D., et. al	ImmunoglobulinG4‐related sclerosing disease of the paranasal sinuses: A case report and literature review	46, M	Ethmoid sinus extending to frontal recess
14.	Gorostis, S., et al	Right ethmoid eosinophilic angiocentric fibrosis with orbital extension	61, M	Orbital mass extending to ethmoidal air cells and optic nerve
15.	Johal, K., et al	Immunoglobulin G4 sinusitis in association with aspirin‐exacerbated respiratory disease	61, M	Nasal polyps extending to ethmoid roof, all sinuses and skull base
16.	Bashyam, A., et al	Immunoglobulin G4‐related disease of the paranasal sinuses	71, F	Maxillary antrum extending into the nasal cavity
17.	Detiger, S. E., et al	IgG4‐Related Disease of Skull Base: Case Series of 3 Patients with Headache	73, M (other two patients were exclusive skull base lesions)	Nasopharyngeal mass
18.	Chowsilpa, S., et al	Temporal bone involvement of IgG4‐related disease: a rare condition misleading to petrous apicitis causing lateral rectus palsy	19, F	Maxillary sinus, ethmoid sinus, sphenoid sinus. Temporal bone and middle cranial fossa extension
19.	Rodriguez Fonseca, O. D., et al	Isolated Immunoglobulin G4–Related Disease of Nasal Septum and Maxilla	52, F	Maxillary sinus, hard palate, and alveolar process
20.	Jurkov, M., et al	IgG4‐related orbitopathy as an important differential diagnosis of advanced silent sinus syndrome	77, M	Paranasal sinus and orbit
21.	Ueno, M., et al	Five Cases of IgG4‐related Disease with Nasal Mucosa and Sinus Involvement	69, F x 264, F41, F39, F	Lacrimal gland, parotid gland and submandibular gland swelling, sinus congestion

## 5 CONCLUSION

We hope this case report promoted broader recognition of IgG4‐RD among physicians and researchers, also highlighting the extremely rare manifestation of IgG4‐RD in the sino‐nasal cavity. While ruling out malignancy, lymphoma and infection are critical before considering IgG4‐RD, and investigations such as histopathology and serology should not be delayed maximizing patient outcome given studies have shown good prognosis with early intervention. In terms of demographic, it would be interesting to learn more about patient cases from different parts of Asia who may contribute to this reported predisposition for head and neck manifestations of IgG4‐RD.^16^, ^17^

## CONFLICT OF INTEREST

None declared.

## AUTHOR CONTRIBUTIONS

HAS and MZS: involved in manuscript writing. KS, AAA, and AS: involved in data collection and manuscript writing.

## PATIENT CONSENT

Consent obtained from the patient is available upon request.

## Data Availability

All data generated or analyzed during this study are included in this published article.
